# *JianPi HuaZhuo XingNao* formula (Chinese herbal medicine) for the treatment of minimal hepatic encephalopathy: a protocol for a randomized, placebo-controlled pilot trial

**DOI:** 10.1097/MD.0000000000010526

**Published:** 2018-04-27

**Authors:** XiaoKe Li, DaNan Gan, Ying Li, Peng Zhang, ZhiGuo Li, HongBo Du, LuDan Zhang, Yuan Cheng, YaQiang Zhang, YiJun Liang, YongAn Ye

**Affiliations:** a Department of Gastroenterology, Dongzhimen Hospital affiliated to Beijing University of Chinese Medicine (BUCM); b Institute of Liver Diseases, BUCM; c Beijing University of Chinese Medicine, Beijing, China.

**Keywords:** *JianPi HuaZhuo XingNao* formula, minimal hepatic encephalopathy, traditional Chinese medicine

## Abstract

**Background:**

Minimal hepatic encephalopathy (MHE) is a subclinical state of hepatic encephalopathy with the possibility of developing into overt hepatic encephalopathy (OHE) and having adverse outcomes. However, no preventative medicine for MHE has been recommended so far. The aim is to evaluate the therapeutic effect of the *JianPi HuaZhuo XingNao* formula (*JPHZXN*) on MHE, specifically whether *JPHZXN* decreases OHE occurrence, through a randomized controlled trial.

**Method:**

Seventy-two patients with MHE are enrolled and allocated in a 1:1 ratio in an experimental group and a control group. *JPHZXN* granules and placebos are dispatched to the experimental group and control group, respectively, for 24 weeks. The primary outcome is the incidence of developing OHE. The secondary outcomes are the patients’ performances in number connection test A and the digital sign test as well as results from the health survey and chronic liver disease questionnaire.

**Results:**

This study will provide proof regarding the therapeutic effect of *JPHZXN* among patients with MHE.

**Conclusion:**

The outcomes could grant clinicians an alternative choice when treating potentially progressive patients with MHE.

## 1 Introduction

### 1.1 Background

Minimal hepatic encephalopathy (MHE) is a unique disease stage of covert hepatic encephalopathy (CHE), and it is one of the complications caused by liver dysfunction.^[1]^ As it is in a subclinical state, it is difficult to identify, and it has no apparent psychologic or neurologic clinical manifestation. However, symptoms can be detected through sophisticated neuropsychologic and neurophysiologic tests.^[2]^ MHE debases patients’ quality of life by, for example, restricting their abilities to drive and work, and it is highly possible that MHE will further develop into overt hepatic encephalopathy (OHE).^[3]^ Therefore, MHE poses a significant potential hazard to individuals and society.^[4]^ Thus, it is significant to diagnose MHE and CHE and to prevent MHE from progressing to OHE. According to epidemiologic surveys, MHE or CHE occurs in 20% to 80% of patients with cirrhosiss.^[5–8]^ Therapies including lactulose, lactitol, probiotics, and rifaximin have been applied in the treatment and research of MHE, and they have shown the effect of preventing OHE from the first attack.^[9,10]^ However, none of these therapies is adequate for recommendation as a routine or preventive treatment. According to the 2014 Practice Guideline by the American Association for the Study of Liver Diseases, routine or preventive medicine is not recommended for MHE.^[11]^ The medical community lacks a clear consensus regarding how to treat the disease.

Liver cirrhosis is the most critical cause of hepatic decompensation, and chronic hepatitis B (CHB) is a principal cause of cirrhosis in China, which is a mid-high endemic region.^[12]^ The cure for hepatic encephalopathy, as an essential complication of cirrhosis, shall be systematic and multidimensional. The treatment for CHB is as necessary as the treatment for MHE itself. The *JianPi HuaZhuo XingNao* (*JPHZXN*) formula adopted in this research was refined from therapeutic drugs for CHB used in former studies (the *Shuanghu qinggan* granule and *Yigan yiqi jieyu* granule),^[13]^ having a defined effect for both the treatment of CHB and hepatic encephalopathy. In the situation with the lack of agreement regarding the treatment of MHE, the use of the *JPHZXN* formula with high-risk patients suffering from MHE that originated from CHB-induced cirrhosis can restrict primary diseases and reduce the possibility of OHE attacks. Moreover, the therapy avoids overtreatment by combining CHB and MHE therapies into a single formula of traditional Chinese medicine.

### 1.2 Objectives

#### 1.2.1 Primary objective

The primary objective is to determine whether the *JPHZXN* formula could decrease the occurrence of OHE among patients with MHE.

#### 1.2.2 Secondary objectives

The secondary objectives are to determine whether the *JPHZXN* formula could improve the performance in number connection test A (NCT-A) and the digital sign test (DST) as well as the quality of life of patients with MHE (evaluated by the Medical Outcomes Study item short-form health survey—36 items [SF-36] and chronic liver disease questionnaire [CLDQ]).

### 1.3 Trial design

We designed this trial as a double-blinded, randomized, and controlled exploratory trial. We will implement simple randomization with a 1:1 allocation ratio in 72 eligible patients. The primary endpoint is the occurrence of OHE within 24 weeks.

## 2 Methods: recruitment, interventions, and outcomes

### 2.1 Study setting

Seventy-two patients from the clinic and inpatient department of the Dongzhimen Hospital affiliated to Beijing University of Chinese Medicine, Beijing, are recruited for this pilot study.

### 2.2 Criteria

All patients should provide a signed informed consent form before enrollment.

#### 2.2.1 Inclusion criteria

Participants should meet all the following criteria:

1. Be diagnosed with CHB, referring to the guideline of prevention and treatment for CHB (2015 version)^[14]^
2. Be diagnosed with hepatocirrhosis through a liver ultrasound, computed tomography scan, magnetic resonance imaging, or other imaging examination or show histologic evidence of hepatocirrhosis (including stage 4 liver fibrosis)
3. Be diagnosed with MHE through neurophysiology and neuropsychology tests
4. Be voluntary participants in this project after signing the informed consent form

#### 2.2.2 Exclusion criteria

Participants who meet any of the following criteria should not be enrolled:

1. Are aged under 18 or over 75
2. Have ruled out HBV infection as the primary cause of the hepatocirrhosis
3. Have severe complications of hepatocirrhosis (electrolyte disorders, gastrointestinal bleeding, infections)
4. Have been diagnosed with a mental illness or cerebrovascular disease
5. Have taken a sedative in the last 4 weeks
6. Have been long-term drinkers for at least 5 years (equivalent to 40 g of ethanol per day for males or 20 g/d for females) or heavy drinkers (equivalent to 40 g of ethanol per day) within 2 weeks.
7. Have body temperatures over 37.5°C
8. Have cardiovascular, pulmonary, renal, endocrine, nerve, and hematology diseases
9. Have been diagnosed with a malignant tumor
10. Cannot cooperate with this research due to other reasons

### 2.3 Research personnel

Experienced physicians (attending physicians, with a minimum 5 years of clinical experience in the department of hepatology or gastroenterology) will be responsible for supervising the recruitments and performing host interviews and physical examinations. Postgraduate students who majored in hepatology will be trained by the principal investigator before participating in the study. They will dispatch drugs, fill in case report forms, and input electronic case report forms.

### 2.4 Interventions

#### 2.4.1 Trial drugs

Both groups will be treated with trial drugs for 24 weeks. The experimental group will receive 1 dose per day of the *JPHZXN* formula, whereas the control group will be given the *JPHZXN* formula placebo. The real drugs and placebos are consistent in shape, color, and taste.

The patients will be allowed to continue their basic treatment for hepatocirrhosis and CHB. In addition to receiving the trial drugs for MHE (Detailed in *2.4.4 Relevant interventions*).

The major ingredients of the *JPHZXN* formula are listed in Table 1.

**Table 1:**
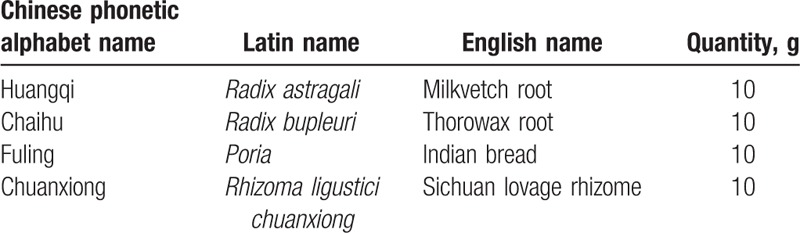
The major ingredients of JPHZXN granule.

#### 2.4.2 Placebos

The corresponding placebos are composed of a mixture of 5% primary formula (trial medicine) with 95% secondary formula (including 0.3% caramel pigment, 0.02% sunset yellow 87, 0.02% tartrazine 85, and 99.66% dextrin).

#### 2.4.3 Criteria for modifying interventions

##### 2.4.3.1 Rescue medicine

Patients who progress from MHE to OHE during the trial will leave the trial cohort and be given standard treatment. They will be given 15 to 30 mL of oral lactulose per dose, with 2 to 3 doses per day. If the condition persists without remission, probiotics and rifaximin will be prescribed. The principles of diagnosis and treatment for OHE abide by the instructions from the American Association for the Study of Liver Diseases.^[11]^

#### 2.4.4 Relevant interventions

##### 2.4.4.1 Basic treatment in addition to the trial drug

Antiviral, antifibrosis, and liver protection therapies are allowed during the course (Table 2), but medications that directly affect blood ammonia or could improve cognitive performance (such as lactulose, lactitol, probiotics, and rifaximin) are prohibited until observable OHE.

**Table 2:**
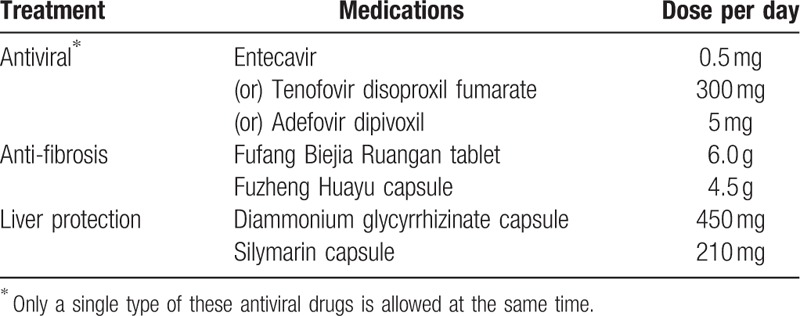
Basic treatments.

### 2.5 Outcomes

The primary therapeutic outcome is the incidence measure of OHE within 24 weeks. An OHE attack is defined an adverse outcome.

The secondary outcomes are the results of the NCT-A, DST, SF-36, and CLDQ. The NCT-A and DST will be applied to measure patients’ neuropsychology performances, and an extended completion time will indicate cognition impairments.^[15,16]^ The SF-36^[17]^ and CLDQ^[18]^ are regular measurements of life quality among patients with hepatocirrhosis, and reduced scores for these tests designate impaired life quality.

### 2.6 Participant time line

**Figure 1:**
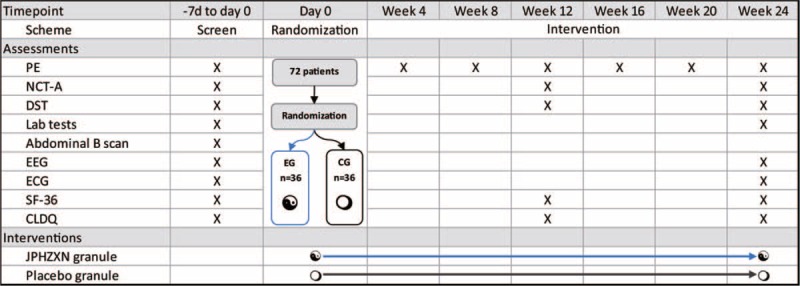
Scheduled intervention and assessments. CLDQ = chronic liver disease questionnaire, DST = digital sign test; laboratory tests include liver functions and blood ammonia, ECG = electrocardiogram, EEG = electroencephalogram, HBV serum markers, HBV DNA, blood routine tests, alpha-fetoprotein, NCT-A = number connection test A, PE = physical examinations, SF-36 = The Medical Outcomes Study item short-form health survey, 36 items.

The enrollment will begin in September and finish by approximately December 2018. The clinical observation will be completed by June 2019. The scheduled timeframe is displayed in Figure 1.

### 2.7 Sample size

Currently, the scientific community has inadequate information about the incidences of OHE that developed from MHE within a specified duration. In this pilot study, we set the sample size to 36 for each group, with 72 participants in total.

### 2.8 Recruitment

Internal physicians will screen eligible patients from the clinic and inpatient department. The principal investigator will introduce the protocol as well as the benefits and risks of the study to the participants. An informed consent form is mandatory for enrollment.

## 3 Method: assignment of interventions

### 3.1 Allocation concealment

The ethics committee will generate a random number table (containing the patients’ IDs) using Microsoft Excel to settle the grouping plan; each group will be assigned 36 IDs, which will be in a disorganized sequence. Each eligible patient will be given a sealed envelope with an ID after enrollment.

### 3.2 Blinding

The interviewers and patients will both be blinded to the grouping information of each participant. The trial drugs and placebos are consistent in color and taste. The ethics committee and data management committee will keep the allocation sequence and grouping information, which they will not release until final analysis or emergency unblinding.

## 4 Method: data collection, management, and analysis

### 4.1 Interviews

Patients will be interviewed every 4 weeks after enrollment in the clinic, where they should return the used packages and remaining drugs. Adequate trial drugs for 28 days will be dispatched during each interview. A series of tests will be conducted in different weeks (Fig. 1).

### 4.2 Data management

Information obtained from each visit and the laboratory results will be recorded on printed case report forms. Electronic data will be manually inputted into double entries in electronic case report forms generated by EPIDATA software. Case report forms and data sets will be stored in the local archive center at Dongzhimen Hospital.

### 4.3 Statistical methods

We will carry out efficacy analyses with the full analysis set, which will include those who provided baseline data and at least one post-treatment assessment. Safety analyses will assess all of the randomly assigned patients.

We will apply a chi-square test to compare the occurrence of OHE attacks among groups.

We will assess the secondary outcomes with the Shapiro–Wilk test. We will assess the normally distributed data with *t* tests. We will assess data that were not normally distributed with nonparametric Wilcoxon rank-sum testing.

## 5 Methods: monitoring

### 5.1 Data monitoring

The Centre for Evidence-Based Chinese Medicine at the Beijing University of Chinese Medicine will commit to the data monitoring—including recruitment supervision, allocation administration, integrity checks of the case report forms, and the overseeing of the unblinding. Interim analysis is not applicable for this pilot study.

### 5.2 Safety monitoring

At each visit, physicians will document any adverse events. When severe adverse events occur or the patient suffers from progressive OHE and needs intensive medical care, the event will be reported to the ethics committee and data management committee to determine whether an emergency unblinding is necessary for the patient. The data management committee is also authorized to perform the unblinding, access the final data set, and evaluate the outcomes.

## 6 Research ethics and dissemination

### 6.1 Ethics approval

The ethics committee of Dongzhimen Hospital, affiliated to Beijing University of Chinese Medicine, approved this protocol on August 8, 2017 (DZMEC-KY-2017-23).

### 6.2 Protocol amendments

The principal investigator is responsible for presenting protocol modifications to the ethics committee and data management committee before the modifications are implemented. The ethics committee and data management committee should agree upon significant changes to the protocol—including changes to the objectives, sample size, interventions, and study procedures—before their implementation. We will formally document minor changes—such as corrections of typos and clarifications—in the memorandum.

### 6.3 Confidentiality

We will write the names of all the participants in abbreviated Pinyin on the case report forms. The participants’ identities, personal information, and medical records obtained from the study are confidential and can only be accessed by the data management committee and ethics committee when necessary. Examined laboratory specimens will be destroyed following clinical regulations (30 days after collection). Informed consent forms, which contain the patients’ signatures, will be stored separately in the archive center.

### 6.4 Policies of data sharing and distributions

During the trial, only the data management committee will be authorized to access the full data set (including the grouping information). The principal investigator is allowed to view all intrastudy medical records but not the allocation data. The ethics committee can acquire the patients’ information only if a severe adverse event occurs and premature unblinding is necessary.

The patients’ ID numbers and medical records will be available from the study leader upon reasonable request after the study is complete. Each patient will obtain a copy of his/her case report form after the unblinding.

## 7 Discussion

Research on the treatment of MHE through traditional Chinese medicine is insufficient. With the features of treatment being based on symptom differentiation, traditional Chinese medicine therapy can act as an early intervention in diseases with insidious manifestations (or subclinical diseases) such as MHE, but the evidence does not sufficiently address the lack of consistent diagnostic criteria and has the confirmation of a randomized placebo-controlled trial. We believe this pilot trial might provide a promising approach to treating CHB-induced MHE in antiviral aspects and preventing OHE.

## Acknowledgments

The authors thank Professor Jianping Liu and Huijuan Cao, from The Centre for Evidence-Based Chinese Medicine at the Beijing University of Chinese Medicine for valuable discussion.

## Author contributions

YAY, XKL, and DNG conceived the study. XKL presided the project. ZGL, LDZ, YC, YQZ, YJL, and HBD helped with the implementation. YL hosted statistical support. PZ will conduct the primary statistical analysis. All authors contributed to the refinement of the study protocol and approved the final manuscript. XKL is the principal investigator, DNG, ZGL, YC, and YQZ are research physicians. HBD, YAY, YL, and PZ are Data Management Committee (DMC) members.

**Roles and responsibilities**

**Principal investigator and research physician**

Design the protocol

Enroll patients

Prepare case report forms

Perform the clinical interview

Dispense the trial drug

Publish the protocol and reports

**Ethics Committee**

Agree on the final protocol

Approve significant amendments to the protocol

Determine the allocation plans by generating the random number table

Agree on suspending the study and prematurely unblinding

**Data management committee**

Design case report form and electronic case report form

Supervise the recruitment

Administer the allocation

Oversee the unblinding

Data management will be executed by the Centre for Evidence-Based Chinese Medicine, Beijing University of Chinese Medicine.

**Data curation:** Ying Li.

**Formal analysis:** Ying Li, Peng Zhang.

**Investigation:** DaNan Gan, ZhiGuo Li, HongBo Du, Yuan Cheng, YaQiang Zhang.

**Methodology:** DaNan Gan.

**Project administration:** XiaoKe Li.

**Supervision:** XiaoKe Li, YongAn Ye.

**Writing – original draft:** XiaoKe Li.

## References

[1] Gerber T Schomerus H Hepatic encephalopathy in liver cirrhosis: pathogenesis, diagnosis and management. Drugs 2000;60:1353–70. 11152016 10.2165/00003495-200060060-00008

[2] Tapper EB Parikh ND Waljee AK Diagnosis of minimal hepatic encephalopathy: a systematic review of point-of-care diagnostic tests. Am J Gastroenterol 2018;113:529–38. 29533396 10.1038/ajg.2018.6

[3] Lewis JH Stine JG Review article: prescribing medications in patients with cirrhosis – a practical guide. Aliment Pharmacol Ther 2013;37:1132–56. 23638982 10.1111/apt.12324

[4] Quero Guillén JC Groeneweg M Jiménez Sáenz M Is it a medical error if we do not screen cirrhotic patients for minimal hepatic encephalopathy? Rev Esp Enferm Dig 2002;94:544–57. 12587235

[5] Romero-Gómez M Córdoba J Jover R Value of the critical flicker frequency in patients with minimal hepatic encephalopathy. Hepatology 2007;45:879–85. 17393525 10.1002/hep.21586

[6] Bajaj JS Management options for minimal hepatic encephalopathy. Expert Rev Gastroenterol Hepatol 2008;2:785–90. 19090738 10.1586/17474124.2.6.785

[7] Sharma P Sharma BC Puri V Critical flicker frequency: diagnostic tool for minimal hepatic encephalopathy. J Hepatol 2007;47:67–73. 17459511 10.1016/j.jhep.2007.02.022

[8] Wang JY Zhang NP Chi BR Prevalence of minimal hepatic encephalopathy and quality of life evaluations in hospitalized cirrhotic patients in China. World J Gastroenterol 2013;19:4984–91. 23946605 10.3748/wjg.v19.i30.4984 PMC3740430

[9] Sharma P Sharma BC Agrawal A Primary prophylaxis of overt hepatic encephalopathy in patients with cirrhosis: an open labeled randomized controlled trial of lactulose versus no lactulose. J Gastroenterol Hepatol 2012;27:1329–35. 22606978 10.1111/j.1440-1746.2012.07186.x

[10] Sidhu SS Goyal O Mishra BP Rifaximin improves psychometric performance and health-related quality of life in patients with minimal hepatic encephalopathy (the RIME Trial). Am J Gastroenterol 2011;106:307–16. 21157444 10.1038/ajg.2010.455

[11] Vilstrup H Amodio P Bajaj J Hepatic encephalopathy in chronic liver disease: 2014 Practice Guideline by the American Association for the Study of Liver Diseases and the European Association for the Study of the Liver. Hepatology 2014;60:715–35. 25042402 10.1002/hep.27210

[12] Liu J Fan D Hepatitis B in China. Lancet 2007;369:1582–3. 17499584 10.1016/S0140-6736(07)60723-5

[13] Ye YA Tian DL Jiang J Effect of Shuanghu Qinggan Granule and Yigan Yiqi Jieyu Granule plus lamivudine on chronic hepatitis B patients: a randomized double-blind placebo-controlled trial. Chin J Integr Med 2016;DOI: 10.1007/s11655-016-2519-9. [Epub ahead of print]. 10.1007/s11655-016-2519-9 27933509

[14] Hou JL Lai W Chinese Society of Hepatology, Chinese Medical Association; Chinese Society of Infectious Diseases, Chinese Medical Association. The guideline of prevention and treatment for chronic hepatitis B: a 2015 update. Zhonghua Gan Zang Bing Za Zhi 2015;23:888–905. 26739464 10.3760/cma.j.issn.1007-3418.2015.12.002 PMC12677373

[15] Ferenci P Lockwood A Mullen K Hepatic encephalopathy—definition, nomenclature, diagnosis, and quantification: final report of the working party at the 11th World Congresses of Gastroenterology, Vienna, 1998. Hepatology 2002;35:716–21. 11870389 10.1053/jhep.2002.31250

[16] Weissenborn K Ennen JC Schomerus H Neuropsychological characterization of hepatic encephalopathy. J Hepatol 2001;34:768–73. 11434627 10.1016/s0168-8278(01)00026-5

[17] Ware JE SF-36 health survey: manual and interpretation guide. Health Institute 1993.

[18] Younossi ZM Guyatt G Kiwi M Development of a disease specific questionnaire to measure health related quality of life in patients with chronic liver disease. Gut 1999;45:295–300. 10403745 10.1136/gut.45.2.295 PMC1727607

